# Variability in the kinesin light chain 1 gene may influence risk of age-related cataract

**Published:** 2007-06-27

**Authors:** Malin E. Andersson, Madeleine Zetterberg, Gunnar Tasa, Mona Seibt-Palmér, Erkki Juronen, Pait Teesalu, Kaj Blennow, Henrik Zetterberg

**Affiliations:** 1 Institute of Neuroscience and Physiology, Department of Psychiatry and Neurochemistry, the Sahlgrenska Academy at Göteborg University, Göteborg, Sweden; 2 Institute of Neuroscience and Physiology, Department of Clinical Neuroscience and Rehabilitation, the Sahlgrenska Academy at Göteborg University, Mölndal, Sweden; 3 Institute of Biomedicine, Department of Medical Chemistry and Cell Biology, the Sahlgrenska Academy at Göteborg University, Göteborg, Sweden; 4 Department of Human Biology and Genetics, University of Tartu, Tartu, Estonia; 5 Institute of Biomedicine, Department of Clinical Chemistry and Transfusion Medicine, the Sahlgrenska Academy at Göteborg University, Göteborg, Sweden; 6 Eye Clinic of University of Tartu, Tartu, Estonia

## Abstract

**Purpose:**

Kinesin-mediated cargo vesicle transport is fundamental to the maintenance of a proper lens fiber structure, which is essential for the transparency of the lens. Here, we test the hypothesis that the rs8702 polymorphism in the kinesin light chain 1 gene (*KLC1*), previously linked to Alzheimer disease (AD), may play a role in cataractogenesis.

**Methods:**

Patients with nuclear (n=76), cortical (n=154), posterior subcapsular (n=117), and mixed (n=148) cataract as well as 183 controls were analyzed for the *KLC1* rs8702 polymorphism using the dynamic allele-specific hybridization (DASH) technique.

**Results:**

The GG genotype of rs8702 was significantly over-represented among cataract patients as compared to controls (63% versus 52%, respectively, p=0.008) and associated with an age-adjusted odds ratio for cataract development of 1.61 (95% confidence interval 1.12-2.31). This association was not confined to any particular cataract type.

**Conclusions:**

The *KLC1* gene may be a novel susceptibility gene for age-related cataract.

## Introduction

Elongated cells such as lens fibers and neuronal axons are highly dependent on the presence of an intact, stabilizing microtubule system. Further, in order to maintain the homeostasis of macromolecules during development and aging, they require a functional cargo vesicle transport system [1-3]. Kinesin is an ATP-dependent motor enzyme that travels along microtubules in a plus-ended direction and plays a fundamental role in the transport of vesicles, mitochondria, and other organelles to the periphery of the cell [4]. It is composed of two subunits. The first is the kinesin heavy chain protein, which contains the ATP- and microtubule-binding motifs that are essential for transport [4]. The second is the kinesin light chain 1 protein encoded by the *KLC1* gene, previously designated *KNS2* [5]. This component associates with the heavy chain and with the membrane vesicles that are transported along the microtubules [6]. Both of these components are expressed in the lens with amyloid precursor protein (APP) and APP-like proteins [2,7] that function as receptors for anterograde transport of vesicles [6].

Recently, a polymorphism in the *KLC1* gene (rs8702, 56,836G>C), localized in a non-coding region that may regulate alternative splicing of the *KLC1* gene transcript [8], was associated with Alzheimer disease (AD) [5]. Given this association, rs8702 may affect *KLC1* function or be in significant linkage disequilibrium with other functionally important polymorphisms. Here, we hypothesize that rs8702 might affect the risk of age-related cataract.

## Methods

### Patients

After informed consent, patients with age-related cataract and control individuals of Estonian nationality, were recruited from two ophthalmic clinics in the town of Tartu and the South-Estonian area. The study was approved by the Ethical Commission at the University of Tartu, Estonia and the tenets of the Declaration of Helsinki were followed. The patients and controls were interviewed about ethnic background and only individuals whose four grandparents all were native Estonians were included. The type of cataract (nuclear [NC], cortical [CC], posterior subcapsular [PSC], and mixed [MC] cataract) was determined using a biomicroscope and an ophthalmoscope prior to surgery. Secondary cataracts, for example cataract due to trauma or diabetes, were excluded. All persons were interviewed to obtain data on smoking habits and participants were thereafter classified into nonsmokers, current, and former smokers. Both current and former smokers had smoked at least five cigarettes per day for at least five years. The case group included 495 patients; 76 with nuclear, 154 with cortical, 117 with posterior subcapsular, and 148 with mixed opacities. This group had a mean age of 72.0±8.7 years (range 47-93 years), 342 (69%) were women, 70 (14%) were current smokers, and 55 (11%) were former smokers. The control group consisted of 187 individuals without cataract, uveitis, or glaucoma. This group consisted of people with a mean age of 65.8±6.9 years (range 43-90 years) where 132 (72%) were women, 18 (9.8%) were current smokers, and 24 (13%) were former smokers. None of the patients or the controls had overt dementia.

### Genetic analyses

Gene symbols used in this study follow the latest recommendations of HUGO Gene Nomenclature Committee [9]. Genomic DNA was obtained from 100 μl whole blood using GenoPrepDNA Blood kit and DNA MagAttract Kit (Qiagen, Hilden, Germany) with GenoM48 Robotic Workstation (GenoVision, Oslo, Norway). Primers for dynamic allele-specific hybridization (DASH) [10] and for sequence polymerase chain reactions (PCRs) enclosing *KLC1* rs8702 and DASH probes were designed using sequence information deposited in the University of California Santa Cruz (UCSC) genome browser. DNA for DASH analysis was amplified using AmpliTaq Gold® (Applied Biosystems, Foster City, CA) under the optimal conditions: 3.0 mM MgCl_2_, 0.16 pmol/μl forward primer (Biotin-TGA CGG TGA CCT GTT GAC GAA A), 0.64 pmol/μl reverse primer (GAG CAC GTG CGG CAC ATT C; Invitrogen, Carlsbad, CA), and at 52.5 °C hybridization temperature. Genotyping of the single nucleotide polymorphism (SNP) was performed using the C probe CTT GCT CTA AGG CTT AG-rox (MWG Biotech, London, UK).

The accuracy of the DASH method was verified by DNA sequencing of 23 unrelated samples representing all three genotypes. DNA for sequencing was amplified using Taq DNA polymerase (Roche Diagnostics, Mannheim, Germany) under the optimal conditions of 1.5 mM MgCl_2_, 0.4 pmol/μl primers (forward: AGC TGT TCA CTT TGG TAA CAG G; reverse: TGC TAC TGG GGC ATA TCC TAG; Invitrogen, Carlsbad, CA) and 56.4 °C hybridization temperature. PCR products were purified using MicroSpin^TM^ S-300 HR Columns (Amersham Biosciences, Buckinghamshire, England). Sequencing reactions were run in sense and anti-sense direction using cycle sequencing with fluorescent dNTPs (ABI PRISM Big Dye Terminator Cycle Sequencing Ready Reaction Kit 3.1, Applied Biosystems, Foster City, CA). Separation by capillary electrophoresis and detection by laser-induced fluorescence was performed with ABI PRISM 3100 Genetic Analyzer (Applied Biosystems).

### Statistics

Deviation from Hardy-Weinberg equilibrium as well as differences in allele and genotype distributions between groups was assessed by the chi2 statistics using SYSTAT 11.0 (SYSTAT Software GmbH, Erkrath, Germany). Odds ratios and 95% confidence intervals were calculated according to Altman [11]. Age-adjusted odds ratios were calculated by logistic regression using the SAS software package (SAS Institute Inc., NC). Statistical significance was defined as p<0.05.

## Results

Observed *KLC1* rs8702 genotype frequencies did not deviate significantly from the expected frequencies on the basis of observed allele frequencies in either of the cataract and control groups and were thus in Hardy-Weinberg equilibrium. The G allele was significantly over-represented in the cataract group compared with the control group (Table 1). Likewise, there was a higher prevalence of the homozygous GG genotype in cataract patients compared with controls (Table 2). The GG genotype was associated with a significantly elevated odds ratio for cataract of 1.59 (95% confidence interval 1.13-2.25).

**Table 1:** *Kinesin light chain 1* rs8702 allele frequencies for cataract and control groups.

***KLC1* rs8702 allele**	Cataract patients (n=990)*	Controls (n=366)*
C	0.2	0.27
G	0.80**	0.73

The G allele of rs8702 is significantly over-represented in cataract patients as compared to controls. The asterisk indicates that "n" is the number of alleles and the double asterisk indicates that p=0.008, cataract versus controls.

**Table 2:** *Kinesin light chain 1* rs8702 genotype frequencies for cataract and control groups.

***KLC1* rs8702 genotype**	**Cataract patients (n=495)**	**Controls (n=183)**
CC	0.028	0.049
CG	0.34	0.43
GG	0.63*	0.52

The GG genotype of rs8702 is significantly over-represented in cataract patients as compared to controls. The asterisk indicates that p=0.008, cataract versus controls.

When subgrouping the patients and controls according to smoking status, current smokers with cataract had a much higher prevalence of the GG genotype than current smokers without cataract (72% versus 44%, p=0.022), which resulted in an odds ratio for cataract of 3.36 (95% confidence interval 1.15-9.77). The effect of the GG genotype among non-smokers was less pronounced (62% versus 41%, p=0.026, odds ratio 1.56, 95% confidence interval 1.05-2.30) and failed to reach significance in the former smoker group (data not shown).

When sub-grouping the patients according to the specific cataract type, the association between the GG genotype and increased risk of cataract remained for all cataract types (p=0.022-0.026) except for the mixed type (Table 3).

**Table 3:** *Kinesin light chain 1* rs8702 genotype frequencies for different cataract types and the control.

***KLC1* rs8702genotype**	**NC (n=76)**	**CC (n=154)**	**PSC (n=117)**	**MC (n=148)**	**Controls (n=183)**
**CC**	**0.013**	**0.032**	**0.026**	**0.034**	**0.049**
CG	0.32	0.32	0.32	0.38	0.43
GG	0.67*	0.64**	0.65#	0.59	0.52

The GG genotype of rs8702 is over-represented in all cataract types, except MC. The asterisk indicates that p=0.022, NC versus controls, the double asterisk indicates that p=0.022, CC versus controls, and the sharp (hash mark) indicates that p=0.026, PSC versus controls. Abbreviations: NC, nuclear cataract; CC, cortical cataract; PSC, posterior subcapsular cataract; MC, mixed cataract.

Given that the cataract patients were older than the controls (p<0.001), the analyses were repeated using logistic regression analysis adjusted for age. By this approach, the GG genotype was associated with an age-adjusted odds ratio for cataract of 1.61 (95% confidence interval 1.12-2.31, p=0.011).

## Discussion

Kinesin molecular motor proteins generate the movement of vesicles containing a wide variety of materials in neuronal and other cells [1,12] and are also expressed in the lens [2,7]. Further, although much more speculative, there are some biochemical and epidemiological data that support an association between cataract and AD [13], the latter of which may be considered a kinesin-related disorder [6]. Both diseases are characterized by aggregation of damaged proteins [13] and several AD-related proteins such as APP, β-amyloid (Aβ), and presenilin (PS) are expressed in the lens together with the kinesins [7,14-16]. These data with the relatively recent finding of an association between the rs8702 polymorphism in the *KLC1* gene and AD [5] led us to hypothesize that the *KLC1* rs8702 polymorphism might affect the risk of age-related cataract as well.

Indeed, there was a difference in *KLC1* rs8702 allele and genotype frequencies between cataract patients and control individuals, which seemed to be a general finding not dependent on cataract type. Thus, faulty kinesin-mediated cargo transport that is associated with this polymorphism may generally potentiate the lens to known environmental risk factors for cataract, such as smoking [17]. In agreement with this view, a possible gene-environment interaction was detected among current smokers with cataract who had a much higher prevalence of the GG genotype than current smokers without cataract.

The *KLC1* rs8702 allele and genotype frequencies found in this study are similar to those reported in other European populations [5,18]. However, when consulting the SNP database, there seems to be a significant population-dependent heterogeneity in the rs8702 genotype distribution with the highest frequency of the homozygous variant reported in an Asian population (31%). Thus, the effect of rs8702 on cataract risk needs to be examined specifically in non-European populations as well.

There are as yet no reports on cataract phenotypes of the different *KLC1* mutant or knockout mice models. However, these mice either express a truncated KLC1 protein or are heterozygous knockouts [19,20], which, hypothetically, may be sufficient to support lens formation.

Interestingly, the risk-conferring genotype detected here was different from the one observed earlier by Dhaenens et al. [5] for AD and from what we have seen with regard to the influence of the *KLC1* rs8702 polymorphism on cerebrospinal fluid biomarkers of AD [18]. In cataract, the GG, not the CC, genotype was over-represented. If the results by Dhaenens et al. [5] and ours hold true in replication studies, inheritance of the C allele would suggest an increase in the risk of AD, whereas inheritance of the G allele would protect against AD but result in elevated risk of cataract. Accordingly, the *KLC1* rs8702 polymorphism does not explain co-morbidity in AD and cataract but rather the opposite.

Finally, it should be noted that the functional consequences of the rs8702 polymorphism are unknown. The polymorphism is located after exon 12 in a non-coding region that seems to regulate the complex alternative splicing of the 3' end of the *KLC1* gene transcript [8]. Interestingly, GenBank gives two different locations of the SNP: one in an intron and the other in the 3'-UTR of an alternative splice product (data base accession numbers NM_182923 and NM_005552, respectively). It is possible that the polymorphism influences or is in linkage disequilibrium with a polymorphism that influences alternative splicing, perhaps in a tissue-specific manner. It is also possible that rs8702 is in linkage disequilibrium with other polymorphisms in the *KLC1* gene or in a nearby gene. Altogether, our results emphasize the need for further studies to reveal the precise role *KLC1* gene variability in cataractogenesis.
